# Guidelines for recognition of occupational cancers in Korea: the results of scientific review by Korean Society of Occupational and Environmental Medicine (2013–2016)

**DOI:** 10.1186/s40557-018-0223-2

**Published:** 2018-02-12

**Authors:** Jaechul Song, Kuck-Hyun Woo, Yang Ho Kim

**Affiliations:** 1 Hanyang University College of Medicine, Seoul, Korea; 2 Soonchunhyang University College of Medicine, Gumi, Korea; 3 Ulsan University College of Medicine, Ulsan, Korea; 4 Department of Occupational and Environmental Medicine, Hanyang University College of Medicine, 222 Wangsimni-ro, Seongdong-gu, Seoul, 04763 Korea

## Abstract

This thematic collection includes the articles to review eleven occupational cancer related risks or working conditions and to propose the guidelines of S. Korea.

The Ministry of Employment & Labor of Korea (MOEL) has expanded its range of occupational diseases, especially for occupational cancers since July 1, 2013. To have more coverage for workers’ compensation, it includes fourteen carcinogens and twelve work related cancers. In order to receive prompt and fair compensation, injured workers need to have the recognition criteria readily available. However, we do not have enough information about the causative factors of occupational cancers, such as the duration and concentration of exposure. Social consensus for compensation is also insufficient. Thus, a permanent organization for discussing and suggesting guidelines for recognition was formed.

In 2013, the Korean Society of Occupational and Environmental Medicine (KSOEM) configured an expert discussion organization to make guidelines for recognition of workers’ compensation with the support of MOEL. The organization has reviewed domestic and foreign standards, scientific basis, domestic workers’ compensation cases, and exposure data of carcinogens. For 3 years, many experts in the organization have reviewed eleven occupational cancer related risks or working conditions and proposed the guidelines.

This special issue includes the guidelines for recognition of occupational cancers due to benzene, painting works, ionizing radiation, shift work, crystal quartz, hexavalent chrome, formaldehyde (HCHO), ethylene oxide, poly aromatic hydrocarbon (PAH), work related infection of hepatitis B or C virus,) and a review article of ‘Probability of Causation’ used to determine the carcinogenicity of ionizing radiation. Even though some of the guidelines have already been published in previous issues of the Annals of Occupational and Environmental Medicine (AOEM), there is a need to organize them so that people engaged in compensation work or study can see relevant documents in one place. The remaining two articles on asbestos and trichloroethylene (TCE) already published elsewhere will be listed here with links to the KSOEM website.

As a Guest Editor, I appreciate the editorial committee AOEM and KSOEM to consider this publication, the reviewers to grant their precious time and the MOEL to support the organization and researchers.

