# Correction to: Guidelines for recognition of occupational cancers in Korea: the results of scientific review by Korean Society of Occupational and Environmental Medicine (2013–2016)

**DOI:** 10.1186/s40557-018-0235-y

**Published:** 2018-04-23

**Authors:** Jaechul Song, Kuck-Hyun Woo, YangHo Kim

**Affiliations:** 1 Hanyang University College of Medicine, Seoul, South Korea; 2 Soonchunhyang University College of Medicine, Gumi, South Korea; 3 Ulsan University College of Medicine, Ulsan, South Korea; 4 Department of Occupational and Environmental Medicine, Hanyang University College of Medicine, 222 Wangsimni-ro, Seongdong-gu, Seoul 04763 South Korea

## Correction

After publication of the original article [1] the authors flagged that the spelling of author YangHo Kim was incorrect. It is published on the original manuscript as Yang Ho Kim, but should be spelt YangHo Kim.

This has been corrected in the author list of this Correction.
